# The world could end cervical cancer if it tried: an interview with Dr Shobha S Krishnan

**DOI:** 10.4155/fsoa-2018-0074

**Published:** 2018-08-22

**Authors:** Shobha S Krishnan

**Affiliations:** 1 MD, FAAFP, President & Founder of Global Initiative Against HPV & Cervical Cancer (GIAHC), NY, NY 10028, USA

**Keywords:** cervical cancer, GIAHC, HPV, India, vaccines

## Abstract

Shobha S Krishnan is the Founder and President of the Global Initiative Against HPV and Cervical Cancer (GIAHC). She is a board-certified family physician and gynecologist and has been involved in the areas of primary care and women's health for over 30 years. She has extensive experience both nationally and internationally in the field of HPV and HPV-related diseases. Her vast experience in this field has given her a unique perspective on various issues related to HPV and its disease consequences.

She is the author of the national and international award-winning book, *The HPV Vaccine Controversy: Sex, Cancer, God and Politics* (Praeger, 2008). Her book chapter on ‘Challenges in Screening for Cervical Cancer’ was recently published in *Cancer Screening in the Developing World* (University Press of New England, May, 2018).

She is the chair of the HPV and Cervical Cancer Task Force at the American Medical Women's Association. She is also a member on the expert's panel of the American Sexual Health Association, Medical Advisory Board of the National Cervical Cancer Coalition and on the scientific committee at the Medical Women's International Association. In addition, she has served on the sexually transmitted disease research working group at Columbia University (NY, USA) and as a surveillance physician for the Centers for Disease Control.

After completing her residency training, Krishnan went into private practice for 10 years before joining Columbia University, where she worked for several years. In December 2015, she received her master's certification in Non-Profit Leadership and Management from the University of Florida (FL, USA).

## Q Can you tell us what led you to where you are today?

I have had a pretty adventurous career. First, I completed a residency in OBGYN and then in family medicine. Soon after, I realized I could combine the two and focus on women's health. I started off with a private practice in Indianapolis (IN, USA), and then moved to New York (USA), where I was at Columbia University for 13 years focusing on women's health and adolescent medicine. In 2006, a publisher approached me based on my background and work performed in both the developed and developing parts of the world. At the time they asked me to speak about the new Human Papillomavirus Virus (HPV) vaccine, which had become somewhat controversial due to it being a sexually transmitted virus. There seemed to be a lot of promise in the vaccine but because it was being recommended for girls around the age of 11 or 12, parents were concerned it was going to promote promiscuity.

The vaccine was marketed to prevent cervical cancer only, and cervical cancer is one of the biggest killers today in rural parts of the developing world. Cervical cancer rates 50–60 years ago were the same in the USA, but now it is not even on the top 10 list because of our fantastic screening program. However, in the developing world, as simple as a pap test is, it is laborious and not widely available. Therefore, women do not present with the disease until they have symptoms, usually at an advanced stage.

In order to address these important concerns and questions, in 2008 Praeger published my book titled *HPV Vaccine Controversy: Sex, Cancer, God and Politics.*

A Yale professor (CT, USA) contacted me after reading my book and was interested in my experiences. She had a student who was very interested in cultural anthropology and healthcare. As a result of the introduction, we sent this student to India, working with her remotely as a team to see if we could do something about the cervical cancer in a rural community I had visited previously.

While pap smears in the developed world are considered the standard of care, we have other tests available for less fortunate areas. In the developing world, we use a simple method called VIA, ‘visual inspection with acetic acid’, which involves painting the cervix with household vinegar. The nice thing about this method is that we can visualize the cervix with the naked eye and if something abnormal is detected we then take a freezing gun and ‘zap’ the cervix for a few minutes to reduce the chance of further growth. This somewhat crude approach has been shown to reduce morbidity and mortality by nearly 40% according to WHO.

Additionally important is the one-stop screen and treat program is done in the same visit. In rural settings if you send women away and then have them come back for treatment, you can lose more than half of them for follow-up care.

This humble small project, headed by the Yale student armed with a group of community health workers subsequently trained at the cancer center in Mumbai (India), was the beginning of our organization, the Global Initiative Against HPV and Cervical Cancer (GIAHC).

## Q Can you tell us more about the objectives of the GIAHC?

Our mission is to save lives from cervical cancer one woman at a time, one day at a time. Our focus is on building awareness, education, prevention through vaccination, screening and early treatment.

Although we are focused on cervical cancer, we are also educating providers, parents, the public, politicians and the research community that HPV does not just cause cervical cancer, but other cancers in both men and women. By 2020 we believe that in the developed world oropharyngeal cancer in men, caused by HPV, will surpass cervical cancers in women. This is because screening tests for these cancers have not yet been developed. What is more alarming, is that HPV is a virus that is ubiquitous. It is believed that by the age of 50, almost 80–90% of people would have had HPV exposure at some point in their lives. Although 90% of these infections clear up on their own, 10% persist owing to contributing factors such as a weakened immune system and cigarette smoking. These are just some of the other topics GIAHC is trying to educate people about.

In the developing world we also act as a coordinating platform; we identify partners and places where need is high and provide them with tools to develop and build local programs to serve both women and children.

## Q How do the obstacles faced by GIAHC differ between the developed & developing parts of the world?

Cervical cancer is something that we can almost completely eliminate by vaccination and screening. However, in the USA, for example, both the vaccination and screening are covered by most insurance. In spite of this, we continue to have about 12,500 cases annually with a reported 4000 deaths. The key message here is that we should not even have one death, because we have the tools to prevent it.

At GIAHC we believe the root of the problem is education. Core to our efforts is to educate providers to recommend the vaccine to parents and patients and to be able to answer any questions they may have regarding cervical cancer or HPV. When it comes to politicians, we talk about how cost effective the vaccine is in reducing overall healthcare cost and how important it is to both men and women's health.

In terms of the developing world where the health infrastructure is not the best, conducting clinical training and creating local care sites are the two big requirements. We have to have proper diagnostic treatment facilities and affordable therapeutics tailored to the local community. Another issue for clinicians and researchers alike is that globally we do not maintain cancer registries, and only when we have this, will we fully understand the incidence, prevalence and mortality rates.

Focusing on the HPV vaccine itself, specifically in the developing world we have to make it affordable, accessible and acceptable. In the USA the vaccine is USD140 a shot, and is a series of two or three depending on age – that is a lot of money, but it is mostly covered by insurance. In the developing world where insurance systems are poor or not existent, the Global Alliance for Vaccines and Immunization (GAVI) has been able to bring down the price of the vaccine to $9 for a whole series of two shots. Even then there are obstacles: you have to be in a GAVI-eligible country; there are quotas; a series of two to three shots/visits reduces compliance; and storage is a problem because the vaccine requires refrigeration. These are just some of the obstacles we are fighting, and that is not to mention the social and cultural norms and all of the political innuendos that go along with it.

Once again it is important to restate, the biggest obstacle is the fact that we have to educate and advise the public. Educational tools and healthcare materials must be designed by listening to locals, addressing their questions, and working with them to make all materials culturally sensitive and linguistically appropriate. We have to come up with inspirational and aspirational messages that strike a chord with patients and the public like the Lady Ganga story. One of GIAHC's communication strategy is to use film and arts in medicine to change minds and lives.

## Q Can you tell us more about how the Lady Ganga film & the partnership with the Global Film Fund?

Ruth Frazier approached GIAHC regarding her daughter, Michele Baldwin, who eventually becomes Lady Ganga, the focus of the film. Michele's cancer had come back for the third time, and was diagnosed as incurable. With this diagnosis in hand she wanted to do something to raise awareness and support our organization and work. Michele had been to India when she was young and had a passion for the country. Knowing that India had the highest number of cervical cancer cases recorded, she wanted to do something. Her intention was to paddle board from the foot hills of the Himalayas all the way to Varanasi to raise awareness of cervical cancer while a friend filmed the journey. This amazing journey was made into a short film for educational use.

It was shown in remote area of India, including a place called Ladakh where a teacher called Nilza saw it and showed it to her friends. The result was just what Michele had envisioned. The group went to their local camp for cervical cancer screening, where, in 3 days, 1700 women were screened and treated. Nilza was diagnosed with cervical precancer. She was treated in a simple procedure, and her story was later added to Michele's original story. The film, Lady Ganga: Nilza's story was later screened on World Cancer Day 2016 at the United Nations in New York City (USA).

Today GIAHC's goal is to help the Global Film Fund (GFF) take the original English film version and  dub it in various languages. Although currently there are some translations available, in the written form, this is not ideal for the developing world. So today we are working with GFF to dub the film in as many languages as possible. We started with Indian languages and are also planning to work on languages commonly spoken in Latin and South American countries.

## Q What have you got on the horizon that we should be looking out for?

The next thing to focus on would be the HPV vaccine. How do we make the vaccine available to people who need it the most? This is not only about education, but are we able to bring the price of the vaccine down? For example, the hepatitis B vaccine was over $100 a shot in the USA and then, albeit 20 years later, it came down to <$1 a shot. Now the number of cases of hepatitis B are very low. We need to do the same for HPV. GIAHC and its partners are looking for ways of making this vaccine affordable, acceptable and accessible to all, both men and women, all over the world.

The other thing we are also interested in is self-sampling. Another good example is colon cancer. Often, getting people to attend colon cancer screening is difficult. Now there is a home test where you send a sample of your fecal material through the mail and if the tests positive it gives you the incentive to get more extensive screenings done. This approach would be really useful for cervical cancer and HPV screening; especially in remote rural areas.

GIAHC and the American Medical Women's Association have partnered to kick off 2019 with a January ‘HPV and Cervical Cancer Awareness Month’ webinar series called: USA vs HPV. This 5-day series is aimed at educating professional and the public on various topics related to cervical cancer and HPV. The program will feature several thought leaders in this field. Also, the Medical Women's International Association is celebrating their centennial in July 2019. We are hoping this conference in NYC will be a good platform to provide educational materials to doctors from all around the world, including regions where cervical cancer cases are high. We are looking forward to this association event in the hope of exchanging knowledge and new ideas so we can end cervical cancer on a global level.

